# Emergence of Plasmids Co-Harboring Carbapenem Resistance Genes and *tmexCD2-toprJ2* in Sequence Type 11 Carbapenem Resistant *Klebsiella pneumoniae* Strains

**DOI:** 10.3389/fcimb.2022.902774

**Published:** 2022-05-12

**Authors:** Xi Li, Weizhong Wang, Xi Jin, Xiaofan Zhang, Xuehan Zou, Qiang Ma, Qingfeng Hu, Haijun Huang, Yuexing Tu

**Affiliations:** ^1^ Laboratory Medicine Center, Department of Clinical Laboratory, Zhejiang Provincial People’s Hospital, Affiliated People’s Hospital, Hangzhou Medical College, Hangzhou, China; ^2^ Department of Respiratory Medicine, Yuhang Second People’s Hospital, Hangzhou, China; ^3^ Department of Infectious Diseases, Zhejiang Provincial People’s Hospital, People’s Hospital of Hangzhou Medical College, Hangzhou, China; ^4^ Department of Critical Care Medicine, Tongde Hospital of Zhejiang Province, Hangzhou, China

**Keywords:** CRKP, *tmexCD2-toprJ2*, *bla*_NDM-1_, plasmids, carbapenem resistance

## Abstract

**Objectives:**

To characterize two plasmids co-harboring carbapenem resistance genes and *tmexCD2-toprJ2* in carbapenem-resistant *Klebsiella pneumoniae* (CRKP) strains.

**Methods:**

Two clinical CRKP strains were isolated and characterized by antimicrobial susceptibility testing, conjugation assays, whole-genome sequencing, and bioinformatics analysis.

**Results:**

The two CRKP strains NB4 and NB5 were both resistant to imipenem, meropenem and tigecycline. Whole-genome sequencing revealed that two CRKP strains belonged to the ST11 type and carried multiple resistance genes. The *tmexCD2-toprJ2* clusters in both strains were located on the IncFIB(Mar)-like/HI1B-like group of hybrid plasmids, which co-harbored the metallo-β-lactamase gene *bla*_NDM-1_. In addition, the co-existence of *bla*_NDM-1_ and *bla*_KPC-2_ and the presence of *tmexCD2-toprJ2* in CRKP strain NB5 was observed.

**Conclusions:**

In this study, *tmexCD2-toprJ2* gene clusters were identified in two NDM-1-producing CRKP ST11 strains. These gene clusters will likely spread into clinical high-risk CRKP clones and exacerbate the antimicrobial resistance crisis. In addition, we detected the co-occurrence of *bla*_NDM-1_, *bla*_KPC-2_ and *tmexCD2-toprJ2* in a single strain, which will undoubtedly accelerate the formation of a “superdrug resistant” bacteria. Hence, effective control measures should be implemented to prevent the further dissemination of such organisms in clinical settings.

## Introduction

Carbapenem resistance genes have been widely identified in various species of *Enterobacteriaceae*, posing a significant threat, especially in clinical environments. Antimicrobial options for the treatment of carbapenem-resistant *Enterobacterales* (CRE) infections are increasingly limited due to the extensive distribution of CRE and the emergence of mobile colistin resistance (*mcr*) genes (Jiang et al., 2020). Tigecycline (TGC) has been regarded as one of the last resort treatment options for infections caused by CRE. Regrettably, the increasing prevalence of CRE has inevitably resulted in increased use of TGC, accelerating the emergence of TGC-resistant isolates (Wang et al., 2018). Of note, TGC-resistant strains have been increasingly observed in clinics since the new drug was approved in 2005. Currently, TGC resistance occurs in chromosome and plasmid factors in gram-negative bacteria. The overexpression of chromosomal multidrug-resistant efflux pumps, such as resistance nodulation division (RND) pumps, AcrAB-TolC pumps, multidrug and toxic compound extrusion (MATE) pumps, and their regulator factors, or mutations, within ribosomal drug-binding sites are considered to be the most common mechanisms for increasing bacterial drug resistance (Sun et al., 2013). However, a growing concern is that the emergence of TGC resistance genes in plasmids may exacerbate transferable resistance among bacterial species. The plasmid-mediated genes *tet*(X3), *tet*(X4), *tet*(X5), and *tet*(X6), which encode enzymatic inactivation proteins against tigecycline, have been detected in animal and clinical isolates (Bai et al., 2019; Chen et al., 2019a; Chen et al., 2019b; He et al., 2019; Sun et al., 2019a; Sun et al., 2019b; Wang et al., 2019; He et al., 2020).

Recently, a novel plasmid-encoded RND efflux pump, the *tmexCD1-toprJ1* gene cluster, was identified in *Klebsiella pneumoniae* isolates from animals, foods, and humans in China (Lv et al., 2020). Subsequently, its orthologous variants *tmexCD2-toprJ2* and *tmexCD3-toprJ3* were reported in *Raoultella ornithinolytica* and *Proteus mirabilis*, respectively (Wang et al., 2021a; Wang et al., 2021b). Likely originating from *Pseudomonas* spp., *tmexCD-toprJ* gene clusters appear to achieve horizontal transfer using adjacent site-specific integrases that confer multidrug resistance (including tetracycline, eravacycline, quinolones, cephalosporins, and aminoglycosides) (Lv et al., 2020). This gene cluster was mainly carried in *K. pneumoniae* but has also been identified in other clinical CRKP strains (Qin et al., 2021). In addition, these strains have various clone types, such as ST15 (Yang et al., 2021), ST37 (Sun et al., 2020) and ST2667 (Qin et al., 2021). Nevertheless, these gene clusters have rarely occurred in ST11-type CRKP, which is a prevalent clinical CRKP clone in China.

However, we report here two plasmids co-harbouring the *tmexCD2-toprJ2* gene cluster and carbapenem resistance genes in two clinical ST11 CRKP strains.

## Materials and methods

### Bacterial Strains

Based on the surveillance of carbapenem resistance organisms (CRO) from clinical specimens of inpatients, all collected strains were identified by MALDI-TOF technology (bioMérieux, Marcy l’Etoile, France) as well as screened for the *tmexCD-toprJ* gene cluster by PCR and Sanger sequencing. Finally, two CRKP strains NB4 and NB5 showed positive for *tmexCD2*-*toprJ2* gene cluster.

### Antimicrobial Susceptibilities Testing

Antimicrobial susceptibility testing was performed according to the reference Clinical and Laboratory Standards Institute (CLSI) (CLSI, 2020). Broth microdilution method was used to measure MIC values for ceftazidime, cefepime, amoxicillin-clavulanic acid, amikacin, ciprofloxacin, meropenem, ertapenem, imipenem, tigecycline and colistin. The results of MICs were interpreted according to CLSI guidelines (CLSI, 2020), except tigecycline and colistin, for which were interpreted according to European Committee on Antimicrobial Susceptibility Testing (EUCAST) criteria for *Enterobacteriaceae* (http://www.eucast.org/clinical_breakpoints). *E. coli* ATCC 25922 was used as a quality control strain.

### Conjugation and Electroporation Experiments

Conjugation and electroporation experiments were performed according to our previous study (Quan et al., 2017). Briefly, conjugation experiments were performed with *E. coli* J53 (Azi^R^) and C600 (Rif^R^) as the recipient strains. 6h growth cultures of the donor strain and the recipient strains were mixed at a ratio of 1:2 in LB broth, and the mixture was then diluted and spread on a MH agar plate containing tigecycline (0.5 mg/liter) and sodium azide (300 mg/liter) or rifampicin (600 mg/liter) for selecting transconjugants. Plasmid DNA was extracted using a Qiagen plasmid midi kit (Qiagen, Germany), then was transformed into electrocompetent *E. coli* DH5α cells. Luria-Bertani agar plates containing tigecycline (0.5 mg/liter) were used to select the transformants, which were further confirmed by PCR targeting at *tmexCD*2-*toprJ*2 gene cluster, 16S rRNA, and antimicrobial susceptibility testing.

### Whole-Genome Sequencing and Bioinformatics Analysis

The genomic DNA of the *K. pneumoniae* NB4 and NB5 strains was obtained using a QIAamp DNA MiniKit (Qiagen, Valencia, CA, USA) following the manufacturer’s recommendations. The combination Oxford Nanopore (MinION system, Nanopore, Oxford, UK) and Illumina sequencing (NovaSeq system, Illumina Inc, San Diego, U.S.A) were used to achieve the complete chromosomes and plasmid sequences, respectively.

The Illumina reads and Nanopore reads were assembled using the hybird assembly tool Unicycler version 0.4.8 (Wick et al., 2017). Annotation of the plasmid genomes was performed using the RAST annotation website server (http://rast.nmpdr.org/rast/cgi).

Antibiotic resistance genes (ARGs), plasmid replicon types, and sequence type of the strains were obtained by the ResFinder 4.1, PlasmidFinder 1.3 and MLST 2.1 servers, which are available at the Center for Genomic Epidemiology (http://www.genomicepidemiology.org/). The virulence factors were identified using the kleborate software (https://github.com/katholt/Kleborate). BRIG and Easyfig were used to visualize the plasmid comparisons and genetic context comparisons, respectively (Alikhan et al., 2011; Sullivan et al., 2011).

### Nucleotide Sequence Accession Numbers

The complete genome sequences of *K. pneumoniae* NB4 and NB5 reported in the present study were deposited in the GenBank nucleotide database under accession no. CP091986-CP091987, CP091992 and CP092653-CP092656.

## Results

### Characteristics of Two Clinical CRKP Isolates

The two CRKP strains NB4 and NB5 were both isolated from the urine of hospitalized patients in 2017 and displayed an almost consistent susceptibility pattern. They were resistant to amoxicillin-clavulanic acid, cefepime, ceftazidime, ertapenem, imipenem, meropenem, amikacin, ciprofloxacin and tigecycline, but susceptible to colistin (**Table 1**). Whole-genome sequencing analysis showed that the two *K. pneumoniae* strains were classified as sequence type 11 (ST11).

**Table 1:** Antibiotic susceptibility of CRKP strain NB4 and NB5.

Isolates	MICs (mg/L)									
	FEP	IMP	ETP	CAZ	AMK	CIP	MEM	TGC	AMC	COL
NB4	>128	>128	>128	>128	>128	>128	>128	8	>128	0.5
NB5	>128	>128	>128	>128	>128	>128	>128	4	>128	0.5
ATCC25922^a^	0.125	0.25	<0.5	0.125	0.5	0.125	<0.125	0.125	4	<0.25

FEP, cefepime; IMP, imipenem; ETP, ertapenem; CAZ, ceftazidime; AMK, amikacin; CIP, ciprofloxacin; MEM, meropenem; TGC, tigecycline; AMC, Amoxicillin-clavulanic acid; COL, colistin.

Drug susceptibility was determined with broth microdilution method. All susceptibility tests were repeated at least three times according to CLSI method. The results of colistin and tigecycline susceptibility were interpreted according to EUCAST breakpoints.

a Quality control strain of antibiotic susceptibility test.

CRKP strain NB4 carried a 5.30-Mb chromosome and three plasmids. Among three plasmids, the *bla*_NDM-1_ gene was carried on plasmid pNB4_NDM. It carried twenty known antibiotic resistance genes (ARGs), including *bla*_NDM-1_, *bla*_DHA-1_, and *fosA3.* CRKP strain NB5 carried a 5.30-Mb chromosome and three plasmids, such as pNB5_NDM (355.4 Kb) and pNB5_KPC-2 (71.6 Kb). It carried twenty-eight known ARGs, including *bla*_NDM-1_, *bla*_DHA-1_, *bla*_KPC-2_ and *fosA3* (**Table S1**). To the best of our knowledge, we are reporting the co-existence of *bla*_NDM-1_ and *bla*_KPC-2_ and the presence of *tmexCD2-toprJ2* in CRKP for the first time.

BLASTn against the virulence genes database (http://bigsdb.pasteur.fr/klebsiella/klebsiella.html) displayed that two strains carried virulence genes *ipaH* (invasion plasmid antigen) and *acrB* (acriflavine resistance protein B). Virulence plasmid-bearing virulence genes, such as *iro*, *iuc*, *rmpA*/*rmpA2* were not present in the two strains.

### Genetic Context of *tmexCD2*-*toprJ2*-Carrying Plasmids

**Figure 1:**
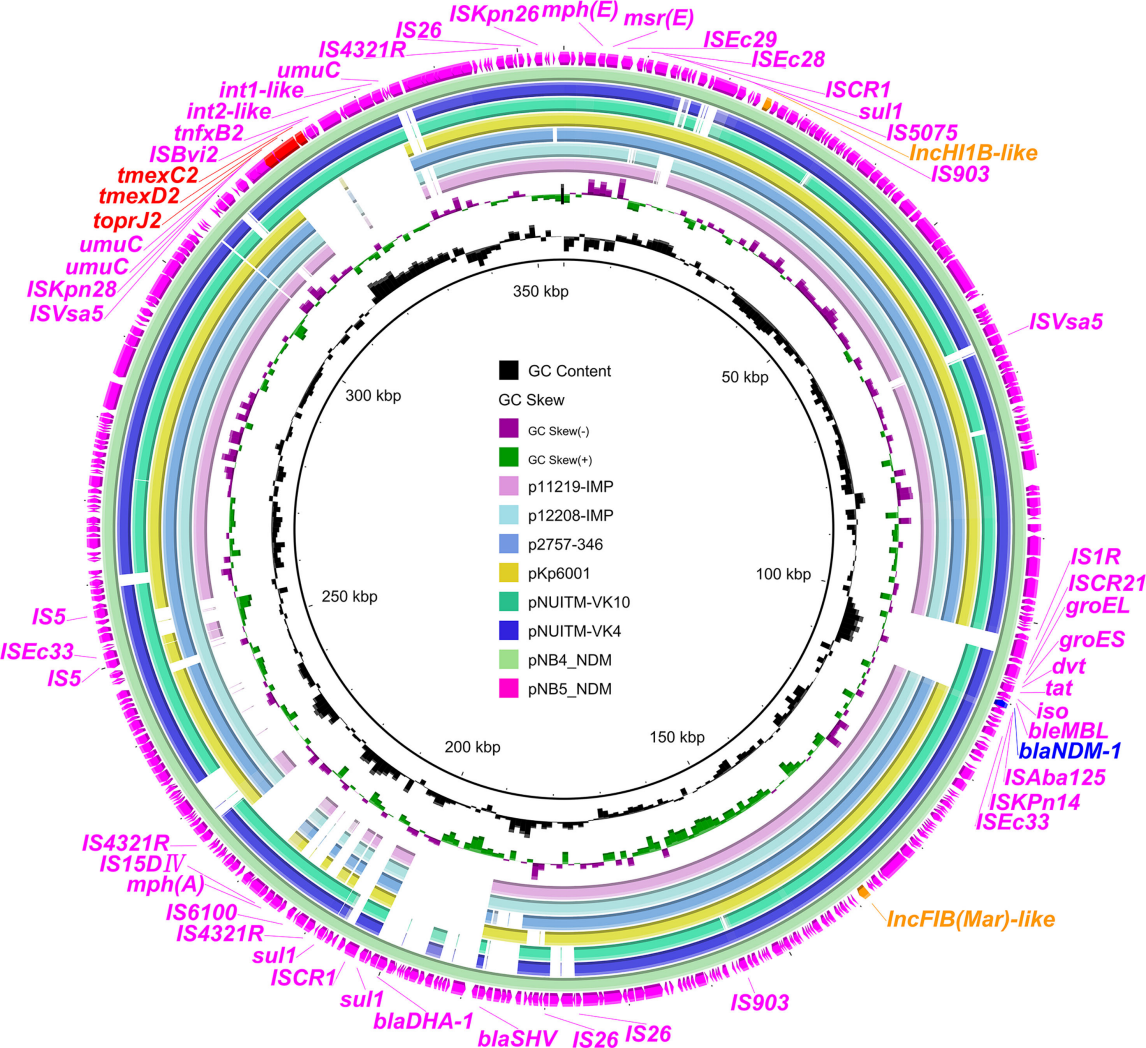
Comparison analysis between *tmexCD2*-*toprJ2*-bearing plasmids and other similar plasmids. The two external rings represent the structures of pNB5_NDM (pink) and pNB4_NDM (aqua). Other similar plasmids were pNUITM-VK4 (GenBank accession no. AP025165.1), pNUITM-VK10 (GenBank accession no. AP025166.1), pKp6001 (GenBank accession no. CP082291.1), p2757-346 (GenBank accession no. CP060810.1), p12208-IMP (GenBank accession no. MF344562.1), and p11219-IMP (GenBank accession no. MF344561.1). The figure was constructed using BRIG.

**Figure 2:**
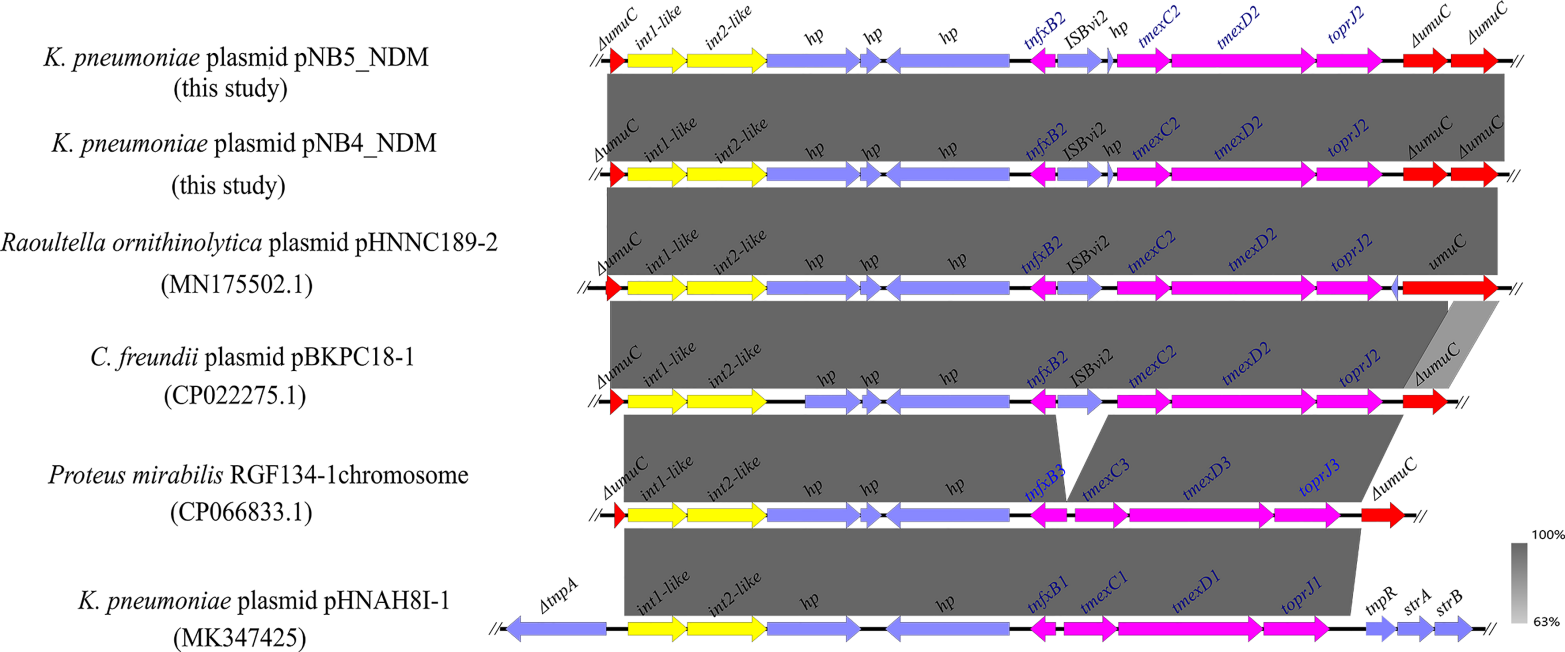
Comparison analysis of the genetic context of *tmexCD2*-*toprJ2* with that of closely related sequences. The pink arrows indicate *tnfxB2-tmexCD2-toprJ2* and *tnfxB1-tmexCD1-toprJ1-like* gene clusters. The yellow arrows indicate the *int-like* genes. The gene *umuC* is indicated by red arrows. IS*Bvi2* and *hp* are indicated by blue arrows. The symbol Δ indicates that the gene is truncated.

To investigate the core genetic environment of *tmexCD2-toprJ2* in the two *K. pneumoniae* strains, two complete *tmexCD2-toprJ2*-carrying plasmids were successfully obtained using a hybrid assembly strategy combining short-read and long-read data. The two *tmexCD2-toprJ2*-carrying plasmids were designated pNB4_NDM in strain NB4 and pNB5_NDM in strain NB5. Both belonged to the IncFIB(Mar)-like/HI1B-like group of multi-replicon plasmids, which were different from the first discovered *tmexCD2-toprJ2*-positive IncFIB_K_ plasmid pHNNC189-2 found in *R. ornithinolytica*. Plasmid sequence comparison showed that pNB4_NDM and pNB5_NDM had highly conserved plasmid synteny and structure, with 100% nucleotide identities (**Figure 1**). Furthermore, the backbones of pNB4_NDM and pNB5_NDM were similar to those of the two *tmexCD2-toprJ2*-carrying plasmids pNUITM-VK4 and pNUITM-VK10 in the nr database, which were harboured by *K. quasipneumoniae* (**Figure 2**). In addition, these two plasmids could not be transferred to recipient cells by conjugation or transformation in *E. coli* J53 *and E. coli* C600 strains after three attempts.

**Figure 3:**
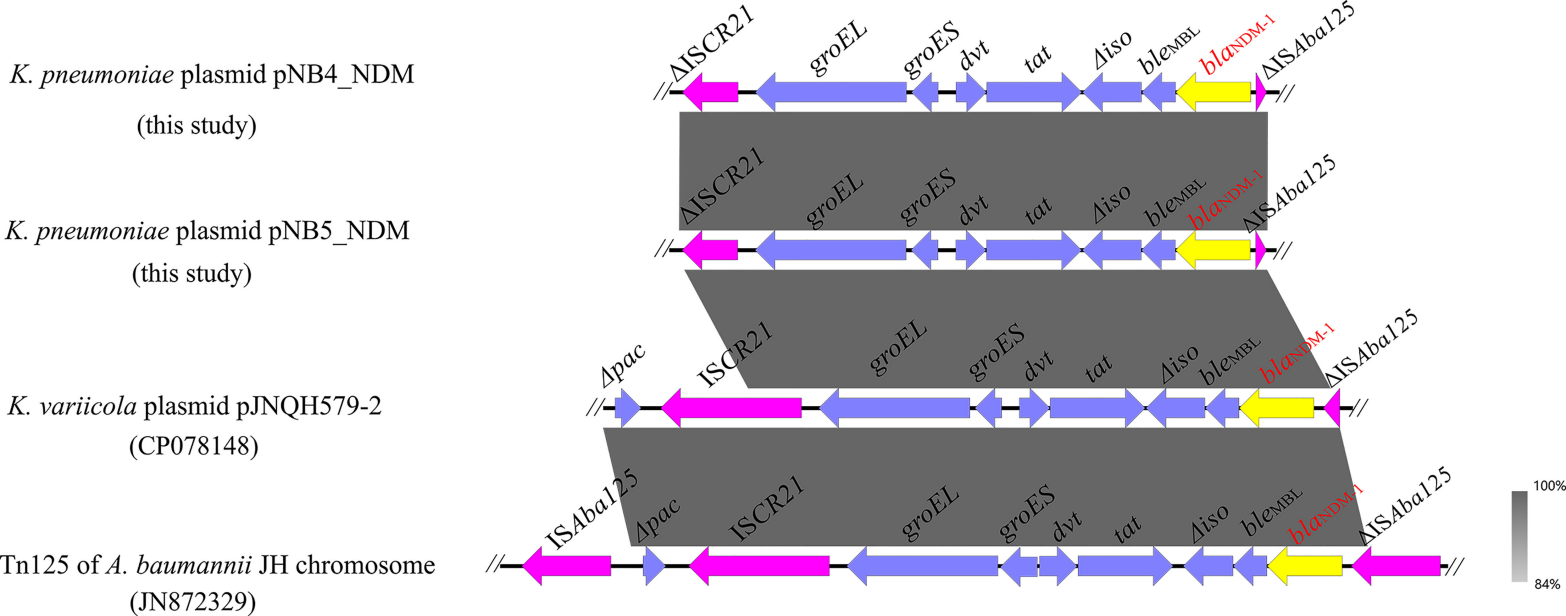
Genetic context of *bla*_NDM-1_ located on plasmids pNB4_NDM and pNB5_NDM compared with other similar sequences. The yellow arrows indicate the *bla*_NDM-1_ genes.

Comparative analysis demonstrated that a similar genetic context like *tmexCD2-toprJ2* was observed in the *tmexCD2-toprJ2*-bearing plasmids pNB4_NDM and pNB5_NDM. Meanwhile, we found that the *tnfxB2-tmexCD2-toprJ2* gene clusters were inserted into the *umuC* gene. A similar structure was also found in the plasmids of *Raoultella ornithinolytica* and *C. freundii* in the nr database (**Figure 3**), which indicated that the two int-like genes may contribute to the mobilization of the *tmexCD2-toprJ2* gene cluster. Moreover, the *umuC* gene was an integration hotspot for the two integrases.

**Figure 4:**
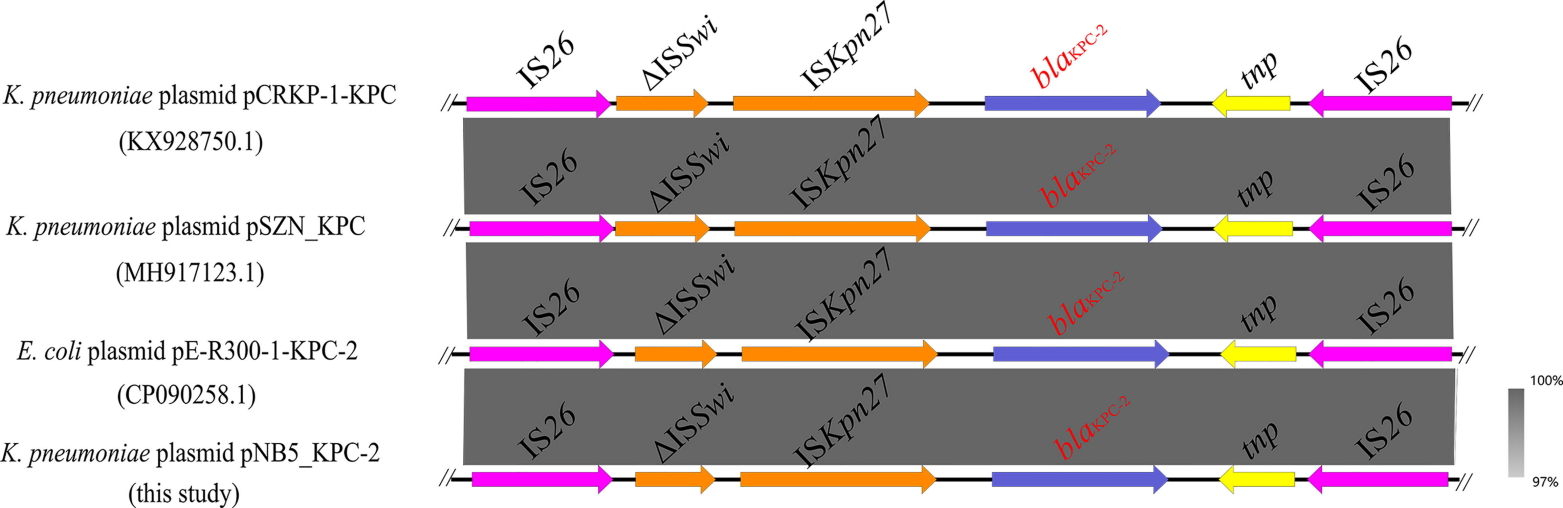
Genetic context of *bla*_KPC-2_ located on *K. pneumoniae* strain NB5 plasmid pNB5_KPC-2 compared with other similar sequences. The blue arrows indicate the *bla*_KPC-2_ genes.

Apart from *tmexCD2-toprJ2*, pNB4_NDM and pNB5_NDM also contained the carbapenemase-encoding gene *bla*_NDM-1_, which was located on the same plasmids as the *tmexCD2-toprJ2* gene cluster. Genetic structure analysis showed that *bla*_NDM-1_ was located in a truncated transposon Tn*125* in plasmids pNB4_NDM and pNB5_NDM (**Figure 4**), with the structure of “ΔIS*Aba125-bla*_NDM-1_ *-ble*_MBL_-Δ*iso-tat-dvt-groES- groEL-Δ*IS*CR21”*. The structure of a truncated transposon Tn*125* containing *bla*_NDM-1_ was also observed in the *tmexCD2-toprJ2*-carrying plasmid pJNQH579-2 (Wang et al., 2021c). Furthermore, we noticed that transposon Tn*125* seems to be the major vehicle for the dissemination of *bla*_NDM-1_ genes in *Klebsiella* spp.

Interestingly, the carbapenemase-encoding gene *bla*_KPC-2_ was also discovered in the IncN/U-type plasmid pNB5_KPC-2 (**Figure 4**). It is worth emphasizing that IS*26*-mediated transmission of the *bla*_KPC-2_ gene has been detected in many strains (**Figure 4**), and it is vital for the dissemination of multiple resistance genes in these bacteria to be monitored closely.

### Genomic Analysis of Plasmids Harbouring *tmexCD*-*toprJ* Gene Clusters

**Figure 5:**
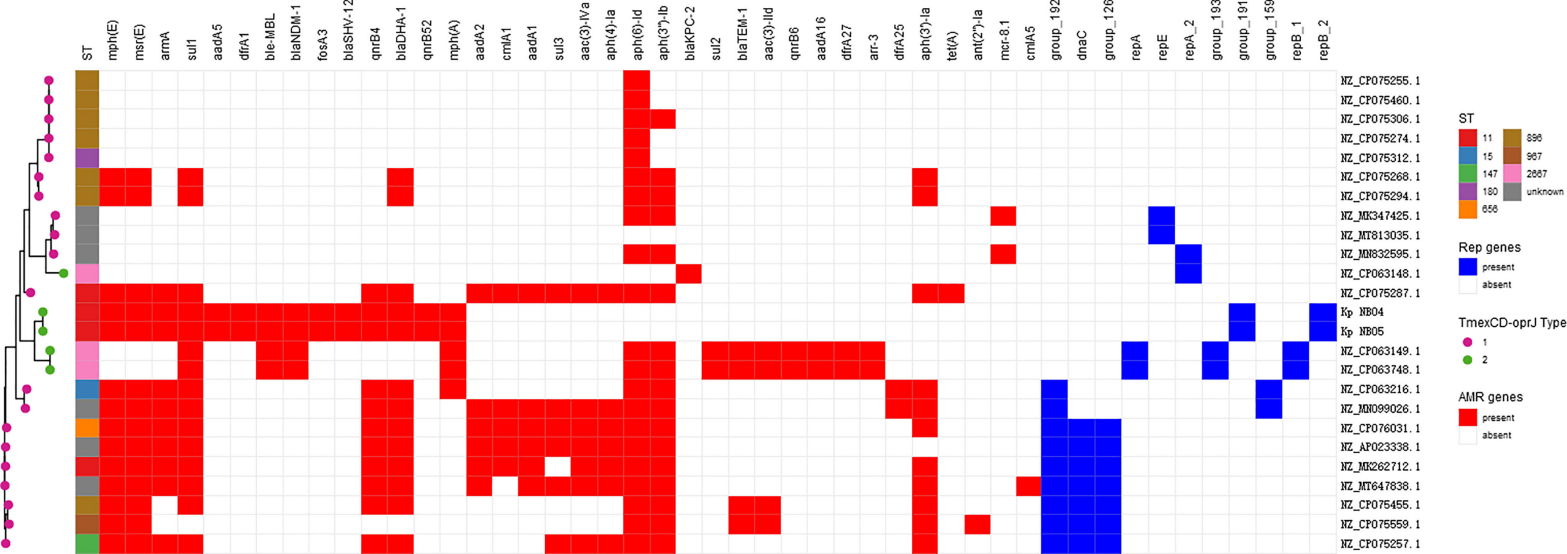
*tmexCD-toprJ*-harboring plasmids in *K. pneumoniae* strain. The heatmap shows the distribution of plasmid replicons (dark blue boxes) and antibiotic resistance genes (red boxes) detected within 25 tmexCD-toprJ-harboring plasmids. The variants of *tmexCD-toprJ* resistance genes are indicated in pink (*tmexCD1-toprJ1*) and green (*tmexCD2-toprJ2*). GenBank accession numbers and species are listed on the right-hand side.

Comparative analysis of the plasmid database revealed that a total of 25 plasmids carried *tmexCD-toprJ* gene clusters in clinical *K. pneumoniae* strains (as of 06 December 2021) (**Figure 5**), 20 strains had *tmexCD1-toprJ1*, 5 strains had *tmexCD2-toprJ2*, and no strain carried *tmexCD3-toprJ3*. Of note, 20% (5/25) of the strains co-harboured carbapenem resistance genes and *tmexCD-toprJ* gene clusters, including 4 strains carrying the *bla*_NDM-1_ gene and 1 strain carrying the *bla*_KPC-2_ gene. In addition, 35% (7/20) of *K. pneumoniae* strains carrying *tmexCD1-toprJ1* gene clusters were ST967, and 60% (3/5) of strains carrying *tmexCD2-toprJ2* gene clusters were ST2667. Sixteen percent (4/25) of the strains carrying *tmexCD-toprJ* gene clusters were ST11, including 2 strains carrying *tmexCD1-toprJ1* gene clusters and the 2 strains carrying *tmexCD2-toprJ2* gene clusters characterized in this study.

## Discussion

Unlike the *tmexCD1-toprJ1* gene, which is primarily found in *K. pneumoniae*, and the *tmexCD3-toprJ3* gene, which is frequently found in *P. aeruginosa* (Wang et al., 2021c), the *tmexCD2-toprJ2* gene was identified among various bacterial species, including *Raoultella ornithinolytica, Citrobacter freundii, Aeromonas hydrophila, K. quasipneumoniae, K. variicola*, and *K. michiganensis* (Wang et al., 2021a; Wang et al., 2021c). Our study further demonstrated that *tmexCD2-toprJ2* spread into CRKP strains, indicating that this gene cluster might have a wider host range than its homologous genes.

Whole-genome sequencing analysis showed that our two CRKP strains both belonged to the ST11 clone type. Currently, clonal spreading is one of the primary modes of CRKP dissemination. In China, ST11-type CRKP is a common clone lineage (Qi et al., 2011) and is frequently associated with a high fate of mortality, posing a severe challenge in clinical treatment (Giacobbe et al., 2015). In this study, we conclude that these two isolates are clonally related based on their identical STs, plasmid components, and resistomes. Our data indicate the potential clonal dissemination of *tmexCD2-toprJ2-*positive *K. pneumoniae*. As a high-risk clinical pathogen to human health, ST11 CRKP strains lack both CRISPR-Cas systems and restriction-modification (RM) systems, which usually have a special ability to acquire resistance genes with high transferability (Liao et al., 2020). We found that *tmexCD2-toprJ2* and *bla*_NDM-1_ were co-encoded by the same plasmids in both the ST11 CRKP NB4 and NB5 strains. Meanwhile, many resistance genes were identified in the two strains. Furthermore, the co-existence of *bla*_NDM-1_, *bla*_KPC-2_, and *tmexCD2-toprJ2* in CRKP was observed for the first time, indicating that ST11 CRKP will likely spread in the clinical environment due to its robust ability for acquiring drug resistance. In addition, comparative analysis of plasmids from the NCBI nr database revealed frequent co-occurrence of *tmexCD2-toprJ2* and carbapenem resistance genes in the same plasmid harboured by CRKP. These findings suggested that such homologous plasmids were adapted by *Klebsiella* spp., which may be a reservoir for multiple resistance genes, such as carbapenem and tigecycline resistance genes. The co-occurrence of *tmexCD2-toprJ2* and *bla*_NDM-1_ in the same plasmid should be considered seriously as a public health concern because the convergence of “mosaic” plasmids can cause both tigecycline and carbapenem resistance. Furthermore, plasmids co-harbouring the *tmexCD1-toprJ1*, *mcr-8* and *bla*_NDM_ genes have been identified (Sun et al., 2020), which will undoubtedly accelerate the formation of a “superdrug resistant”plasmid.

Of note, pNB4_NDM and pNB5_NDM share a similar plasmid backbone. Plasmid replicon analysis showed that pNB4_NDM and pNB5_NDM harboured two conserved replicon genes. In this study, we call them IncFIB(Mar)-like/IncHI1B-like, which were highly homologous to IncFIB (Mar) and IncHI1B plasmids, respectively. The IncFIB(Mar)/IncHI1B-type plasmids were mainly carried by *Klebsiella* spp. in food production chains according to the host range analysis and seemed to be primary vectors for the horizontal dissemination of *tmexCD1*-*toprJ1* among *Klebsiella* spp. (Peng et al., 2021). Notably, IncFIB(Mar)/IncHI1B-type plasmid carrying *tmexCD1*-*toprJ1* gene could not conjugate to *E. coli* J53 and *E. coli* C600 strains but could be transformed to *E. coli* DH5α strains by electroporation (Peng et al., 2021). However, the two plasmids in this study could not be transferred to recipient *E. coli* strains by either conjugation or transformation with *E. coli* strains, indicating that this IncFIB(Mar)-like/IncHI1B-like type plasmids might be more restricted by the host species. Further studies are needed to assess the contribution of two conserved replicons in the host range.

Genetic context analysis showed that *tmexCD2-toprJ2* was in a conserved structure in the two plasmids. An increasing number of studies have revealed that similar structures containing the *tmexCD-toprJ* gene cluster were present in different bacterial species (Wang et al., 2021c). Interestingly, we found that a genetic structure containing *tmexCD2*-*toprJ2* gene clusters and two *int* genes were inserted into the *umuC* gene in both pNB4_NDM and pNB5_NDM plasmids (**Figure 2**). The *umuC* gene appears to be a “hotspot” for *tmexCD-toprJ* clusters integration in chromosomes and plasmids (Peng et al., 2021). The *umuCD* gene was the insertion site of variable region III of SXT/R391 ICEs (Burrus et al., 2006; Carraro and Burrus, 2014), which consisted of many resistance genes such as *tmexCD1*-*toprJ1* (Wang et al., 2021c), t*et(X)* (He et al., 2020; Peng et al., 2020), and *bla*_NDM-5_ (Kong et al., 2020). The prevalence of *umuCD* may play important role in the spreading of the *tmexCD*-*toprJ* gene cluster, while further study is needed to identify this molecular mechanism.

In addition, the *bla*_NDM-1_ carbapenem resistance gene co-existed with *tmexCD2-toprJ2* in both plasmids pNB4_NDM and pNB5_NDM. *bla*_NDM-1_ is in the truncated transposon Tn*125*, which has been identified for years and is widely distributed on multiple plasmids in a variety of bacterial species. More attention is needed to study the genetic structure of *bla*_NDM-1_ driven from Tn*125* to elucidate its possible horizontal transmission mechanisms. The structure of a truncated transposon Tn*125* containing *bla*_NDM-1_ was also observed in the *tmexCD2-toprJ2*-carrying plasmid pJNQH579-2 (Wang et al., 2021c). Furthermore, we noticed that transposon Tn*125* seems to be the major vehicle for dissemination of *bla*_NDM-1_ in *Klebsiella* spp.

## Conclusions

In summary, we report the identification of two clinical *tmexCD2-toprJ2*-encoding ST11 carbapenem-resistant *K. pneumoniae* strains. Dissemination of *tmexCD-toprJ* gene clusters in CRKP strains may pose a substantial threat in clinical treatment settings. TGC has become one of the few therapeutic alternatives against CRKP strains. However, the emergence of TGC resistance gene clusters in CRKP strains carrying *bla*_NDM-1_ or *bla*_KPC-2_ is a matter of major concern because colistin is presently a last-resort antibiotic, alone in its ability to treat infections caused by similar strains. It is essential to continuously monitor such resistance gene clusters in different settings to better understand their specific transmission mechanisms.

## Data Availability Statement

The datasets presented in this study can be found in online repositories. The names of the repository/repositories and accession number(s) can be found in the article/**Supplementary Material**.

## Ethics Statement

This study was conducted in accordance with the Declaration of Helsinki and had been reviewed and approved by the Research Ethics Committee of the Zhejiang Provincial People’s Hospital (QT2022130).

## Author Contributions

Conceived and designed the experiments: QH, HH, and YT; Performed the experiments: XiZ; Analyzed the data: XuZ and QM; Wrote the manuscript: XJ and XL; All authors read and approved the final manuscript.

## Funding

This study was supported by National Natural Science Foundation of China (No. 82172306), Public Technology Research Projects of Zhejiang Province, China (LGD21H190001) the Medical and Health Research Project of Zhejiang Province, China (2020KY420 and 2022KY531) and Health Science and Technology Project of Hangzhou (0020190881).

## Conflict of Interest

The authors declare that the research was conducted in the absence of any commercial or financial relationships that could be construed as a potential conflict of interest.

## Publisher’s Note

All claims expressed in this article are solely those of the authors and do not necessarily represent those of their affiliated organizations, or those of the publisher, the editors and the reviewers. Any product that may be evaluated in this article, or claim that may be made by its manufacturer, is not guaranteed or endorsed by the publisher.
