# Supplementary material for: Emergence of Plasmids Co-Harboring Carbapenem Resistance Genes and tmexCD2-toprJ2 in Sequence Type 11 Carbapenem Resistant Klebsiella pneumoniae Strains
Source: Front Cell Infect Microbiol. 2022 May 12;12:902774. doi: 10.3389/fcimb.2022.902774 (PMC9134201; doi:10.3389/fcimb.2022.902774)
Supplement: Supplementary file 1 [file Table_1.docx]

Table S1. The basic information of strains in this study.

| **Strain IDs** | **ST-type** | **Species** | **Assembly**  **Methods** | **Sequencing platforms** | **No. of**  **plasmids** | **Chromosome or plasmids** | **Size(bp)** | | | **Plasmid type** | **Resistance genes** |
| --- | --- | --- | --- | --- | --- | --- | --- | --- | --- | --- | --- |
| NB4 | 11 | *K. pneumoniae* | Unicycler | MinION, Illumina | 3 | Chromosome | 5,302,095 | | | / | *fosA6*, *bla*_SHV-11_, *oqxA*, *oqxB* |
|  |  |  |  |  |  | pNB4_NDM* | 355,227 | | | IncFIB(Mar)-like, IncHI1B-like | *aadA5*, *armA*, *bla*_DHA-1_, *bla*_NDM-1_, *bla*_SHV-12_, *ble*_MBL_, *dfrA1*, *fosA3*, *mph*(E), *msr*(E), *qnrB4*, *qnrB52*, *sul1*, *tmexCD2-toprJ2* |
|  |  |  |  |  |  | pNB4_2 | 171,032 | | | IncFIB, IncFII | *aph(3')-Ia, mph(*A*)* |
|  |  |  |  |  |  | pNB4_3 | 7,890 | | | Col440I | */* |
| NB5 | 11 | *K. pneumoniae* | Unicycler | MinION, Illumina | 3 | Chromosome | | 5,302,830 | / | | *fosA6*, *bla*_SHV-11_, *oqxA*, *oqxB* |
|  |  |  |  |  |  | pNB5_NDM* | | 355,489 | IncFIB(Mar)-like, IncHI1B-like | | *aadA5*, *armA*, *bla*_DHA-1_, *bla*_NDM-1_, *bla*_SHV-12_, *ble*_MBL_, *dfrA1*, *fosA3*, *mph(*A*)*, *mph*(E), *msr*(E), *qnrB4*, *qnrB52*, *sul1*, *tmexCD2-toprJ2* |
|  |  |  |  |  |  | pNB5_KPC-2 | | 71,683 | IncN, IncU | | *aac(6')-Ib-AKT*, *arr-3*, *bla*_CTX-M-3_, *bla*_KPC-2_,  *bla*_TEM-1_, *dfrA14*, *qnrS1* |
|  |  |  |  |  |  | pNB5_3 | | 171,047 | IncFIB, IncFII | | *aph(3')-Ia*, *mph(*A*)* |

* *tmexCD2-toprJ2* harboring plasmid.
